# Analysis of the Bioprotective Potential of Different Lactic Acid Bacteria Against *Listeria monocytogenes* in Cold-Smoked Sea Bass, a New Product Packaged Under Vacuum and Stored at 6 ± 2°C

**DOI:** 10.3389/fmicb.2021.796655

**Published:** 2021-12-20

**Authors:** Lucilla Iacumin, Giorgia Cappellari, Michela Pellegrini, Marco Basso, Giuseppe Comi

**Affiliations:** Department of Agricultural, Food, Environmental and Animal Science, Università degli Studi di Udine, Udine, Italy

**Keywords:** cold-smoked sea bass, starter, *Listeria monocytogenes*, bioprotection, under vacuum

## Abstract

The aim of the work was to monitor the presence of *Listeria monocytogenes* in cold-smoked fish products (trout, salmon, and sea bass) marketed in Italy. Cold-smoked sea bass is a new product that has not yet been commercialized and was collected from the production facility. Monitoring data have shown that cold-smoked products can be contaminated by *L. monocytogenes*, the presence of which has been highlighted mainly by enrichment culture (presence in 25 g). The isolated *Listeria* were serotyped and belonged mainly to low-virulence serotypes (1/2c), followed by serotypes 1/2a, 1/2b, and 4b. Furthermore, considering the ability of *L. monocytogenes* to grow in these products due to their chemical–physical characteristics (pH > 6.0, Aw > 0.97) and long shelf life at 4°C, an additional aim was to verify the activity of different bioprotective starters, including *Lactilactobacillus sakei* (LAK-23, Sacco srl, *Via* Alessandro Manzoni 29/A, 22071 Cadorago, CO, Italy), *Carnobacterium* spp., *Lacticaseibacillus casei* (SAL 106), and *Lacticaseibacillus paracasei* (SAL 211), in cold-smoked sea bass. All starters were bacteriocin producers. For this experiment, smoked sea bass samples were intentionally inoculated with a mixture of three different strains of *L. monocytogenes* and of each starter culture. After inoculation, the smoked sea bass were vacuum-packed and stored at 6 ± 2°C for 60 days, simulating the typical abuse storage temperature of markets and home refrigerators. At 0, 15, 30, 45, and 60 days, the sea bass samples were analyzed to evaluate the effectiveness of the starters against *L. monocytogenes*. *Listeria monocytogenes* growth was prevented only by the addition of the LAK-23 starter. Indeed, at the end of the shelf life, the amount of *L. monocytogenes* observed was similar to that in the inoculum. Consequently, the use of this starter can allow the inclusion of cold-smoked sea bass or smoked fish products in category 1.3 of Regolamento CE 2073/2005, which are products that do not support the growth of this microorganism. Finally, the activity of the LAK-23 starter did not produce an off flavor or off odor in the smoked sea bass.

## Introduction

Meat from seafood is an important source of high-quality protein for humans (Tidwell and Allan, 2001). However, it is highly susceptible to both microbiological and chemical deterioration due to its high water activity (Aw), neutral pH, relatively high contents of nitrogen compounds and free amino acids and presence of autolytic enzymes (Etemadi et al., 2013). Rapid cooling and storage on ice extend the shelf life of fish and fishery products to only 9–12 days, despite the use of innovative packaging and technological methods (Çoban et al., 2016). To prolong the conservation of fresh fish, technological processes are increasingly used to slow or block degradation activity. Smoking is one of these techniques. Fish products such as salmon and trout are salted and cold-smoked to increase their organoleptic characteristics and shelf lives. Recently, to provide new products with high nutritional value, cold smoking of unconventional fish products has been used. In Friuli, a company expert in the smoking of fish products has studied the potential of cold-smoking sea bass fillets. In Italy, sea bass are usually marketed in boxes with ice flakes or at 4°C packaged in air or under vacuum, both gutted and whole and as fillets. Consequently, their shelf life is limited to 9–12 days. In fact, within a short time, tissue enzymes and contaminating microbial populations adapt to refrigeration temperatures, producing metabolites typical of spoilage, which consist of basic volatile nitrogen (ammonia compounds), trimethylamine, amines, peroxides, and compounds derived from the degradation of lipids (malonaldehyde; Joffraud et al., 2001; Comi, 2016; Iacumin et al., 2017, 2020). Therefore, for trout, salmon and other fish, smoking is increasingly used to extend their shelf lives up to 45–60 days under refrigeration. Sea bass, which are medium-sized fish comparable to trout and salmon, are cold-smoked at a maximum temperature of 29°C. The technology applied is also similar to that used for trout and salmon and includes the selection of raw materials; threading (baffe/fillets); dry salting; traditional cold smoking, without using additives; finishing of the threads; cutting, vacuum packing and storage at 4°C; and delivery and sale of the finished product at 4°C. Therefore, cold-smoked sea bass fillets belong to the category of minimally processed foods, which are foods treated with techniques that preserve them but also retain to a greater extent their nutritional quality, sensory, and hygienic characteristics by reducing the reliance on heat as the main preservative action (Raybaudi-Massilia et al., 2013). Consumers are used to prefer foods which retain their natural nutritional and sensory properties (Sillani and Nassivera, 2015). Indeed the cold-smoked fish are produced by minimal processing techniques that include salting, smoking, vacuum packaging and refrigeration to meet this challenge of replacing traditional methods of preservation whilst retaining nutritional, sensory and hygienic quality.

The microbial ecology of cold-smoked fishery products has been intensively studied and consists of Enterobacteriaceae, *Shewanella putrefaciens*, *Aeromona*s spp., *Pseudomonas* spp., *Photobacterium phosphoreum*, lactic acid bacteria (LAB), and *Brochothrix* spp. (Truelstrup Hansen et al., 1996; Leroi et al., 1998, 2001; Truelstrup Hansen and Huss, 1998; Joffraud et al., 2001, 2006; Cardinal et al., 2004; Lyhs et al., 2007; Bernardi et al., 2009; Leroi, 2010). LAB and *Brochothrix thermosphacta* grow preferable in both vacuum and Modified Atmosphere Packaging smoked fish (Laursen et al., 2005; Leroi, 2010). Unfortunately, also *Listeria monocytogenes* can contaminate cold-smoked fish during the storage. The level of contamination is usually low (<1 CFU/g), but during the 2 month shelf life, it can grow and reaches dangerous loads, as demonstrated by various authors (Guyer and Jemmi, 1991; Ericsson et al., 1997; Ross et al., 2000; Hoffman et al., 2003; Acciari et al., 2017). The cold-smoking process does not kill *L. monocytogenes* (Autio et al., 1999; Miettinen et al., 1999) and the reduced-oxygen atmospheres cannot control the proliferation of psychrotrophic, facultative anaerobic, or strictly anaerobic pathogens, such as *L. monocytogenes*, in fish products (Rutherford et al., 2007; Tosun and Ozden, 2014).

The cold-smoked sea bass are ready-to-eat food, which is a food intended by the producer or the manufacturer for direct human consumption without the need for cooking or other processing effective to eliminate or reduce to an acceptable level the microorganisms of concern (Regolamento (CE) n. 2073/2005, n.d.). This regulation establishes specific microbiological criteria for certain microorganisms for foodstuffs. In particular, Annex I of this regulation sets out the microbiological food safety criteria applicable for *L. monocytogenes* (criteria 1.1 to 1.3), with criterion 1.1 specifically targeting RTE intended for infants and for special medical purposes and the other two criteria (1.2 and 1.3) targeting all other types of RTE foods. When the RTE product can support the growth of the *L. monocytogenes*, the criteria include the absence in 25 g. However, a quantitative limit of 100 CFU/g is set for criterion 1.2 (RTE foods able to support the growth of *L. monocytogenes*) when the manufacturer is able to demonstrate, to the satisfaction of the competent authority, that its product will not exceed the limit 100 CFU/g throughout the shelf-life (AAVV, 2021). The microbial shelf life of RTE corresponds to the period of time during which the food remains within predefined quantitative microbiological limits and it begins from the time the food is produced and/or packed. The cold-smoked sea bass, as all the cold-smoked fish, is a ready to eat food, which for its physicochemical composition can support the *L. monocytogenes* growth, because it does not show pH ≤ 4.4 or Aw ≤ 0.92, or pH ≤ 5.0 and Aw ≤ 0.94. Consequently, its criterion is determined by the absence of *L. monocytogenes* in 25 g product. However, for this product, the manufacturer can request to insert the product in the criterion 1.3 (less than 100 CFU/g product) if it demonstrates to apply a technology able to prevent *L. monocytogenes* growth. The most promising technology could be the use LAB as bioprotective starters’ cultures.

Lactic acid bacteria are considered a new generation of food additives and the basis of food biopreservation (Said et al., 2019). Many studies have been carried out about different RTE food categories that can be contaminated by *L. monocytogenes*. Recent studies have evaluated the ability of LAB to control *L. monocytogenes* in food products such as cheese, sliced cooked cured pork shoulder, diced ham, fresh-cut lettuce, processed seafood, and mayonnaise-based seafood salads, cold-smoked salmon, hot-smoked sea bream, and so on (Bredholt et al., 1999; Mataragas et al., 2002; Allende et al., 2007; Mejlholm and Dalgaard, 2014; Aymerich et al., 2019; Bolìvar et al., 2020; Iacumin et al., 2020; Morandi et al., 2020). In particular, it was observed in sausages, a 1.5–2.5-log reduction of *L. monocytogenes* by the presence of bacteriocin-producing strains belonging to *P. acidilactici*, *L. sakei*, *L. plantarum*, and *L. curvatus* species. These bacteria could be used alone or in combination (Vignolo et al., 2015). LAB are useful because of their antagonistic effect and production of organic acids or bacteriocins against *L. monocytogenes* (Gàlvez et al., 2007; Zhou et al., 2015). These bacteriocin inhibition effects are likely caused by different actions, such as competition for nutrients, as well as organic acid and bacteriocin production, in addition to the “hurdle” parameters. Strains of *Lactobacillus sakei*, *L. casei*, *L. brevis*, *L. curvatus*, *L. plantarum*, and *Carnobacterium* spp. isolated from meat products frequently produce bacteriocins or bacteriocin-like compounds, and in particular, these strains have good antilisterial effects and are therefore used as bioprotective cultures in European meat products (Schillinger et al., 1991; Hugas et al., 1996; Kotzekidou and Bloukas, 1996; Bredholt et al., 2001; Leroy et al., 2005; Vignolo et al., 2015). The main advantage is that LAB inoculation does not influence the quality of the final product and food products do not present any sensory alteration.

Considering that *L. monocytogenes* exhibits different levels of virulence and pathogenicity, several discriminatory typing methods have been described (Brosch et al., 1996; Wernars et al., 1996). Typing by pulsed-field gel electrophoresis (PFGE), which has thus far provided discrimination of strains, has rapidly become the standard typing method for detecting outbreaks of listeriosis (Brosch et al., 1996; Graves and Swaminathan, 2001). However, this method is laborious and time-consuming (Miettinen et al., 1999), lasting 4–5 days. Briefly, it starts by the purified colony and then continues with the preparation and cleavage of genomic DNA in agarose plugs (24 h) and macrorestriction fragments resolved by PFGE (23–30 h) and ends with the visualization by UV transilluminator and photographed (1) of the cut bands (Brosch et al., 1996). Therefore, for practical purposes, it is often preceded by serotyping (Miettinen et al., 1999) which lasts 4 h. Since all major outbreaks of the invasive form of listeriosis are due to serotype 4b strains (Buchrieser et al., 1993; Farber and Harwing, 1996), the procedure adopted for outbreak investigations is based on the characterization of serovars to provide valuable information for rapid screening of strain groups.

Considering the above information, the aim of this work was to monitor the presence of *L. monocytogenes* in different cold-smoked fish and to use bioprotective cultures to eliminate or reduce the growth of *L. monocytogenes* intentionally inoculated into cold-smoked sea bass.

## Materials and Methods

### Monitoring of *L. monocytogenes* in Smoked Fish Products

Approximately 440 samples of smoked fish products were analyzed, including 150 samples of salmon, 140 samples of trout, and 150 samples of sea bass. The salmon and trout samples were collected from supermarkets in Northern Italy, while the smoked sea bass samples were of experimental origin and collected from one facility. Each sample was analyzed for *L. monocytogenes* according to the ISO method (ISO 11290-1:1996 Adm.1:2004, n.d.). In particular, 25 g of product was diluted in Fraser broth (1/2 – Oxoid, Italy). After homogenization, characterization proceeded according to the methods for qualitative or quantitative research (ISO 11290-1:1996 Adm.1:2004, n.d.). From each plate of Agar *Listeria* acc. Ottaviani Agosti (Biolife, Italy), five presumptive *L. monocytogenes* colonies were collected and identified using the same ISO method. In this way, approximately 65 colonies were identified and serotyped according to sera produced by DENKA SEIKEN (Co. Ltd., Tokyo, Japan) distributed in Italy by Biogenetics Diagnostics S.r.l. (Padua, Italy).

### Inhibition of *L. monocytogenes* Intentionally Inoculated Into Cold-Smoked Sea Bass by Different Starter Cultures

#### Substrate Preparation and Group Subdivision

The three lots of sea bass used were raised in sea cages by Orada adriatic d.o.o. in Cres, Croatia. They were collected, eviscerated, placed in polystyrene boxes containing ice, and sent to a processing plant in the Friuli region within 5 h. This company has extensive experience in the cold smoking of fish products such as both farmed and wild salmon and, especially, trout. The fish were filleted (baffe) and salted to a WPS value > or equal to 3.5%, and then they were desalted and smoked at low temperature (<30°C). After smoking, the fillets were vacuum-packed in plastic bags (PE/PA Niederwieser group, Italia), stored at 4°C and transported to the Department of Agricultural Food, Environmental and Animal Sciences of the University of Udine (Di4a). Each sample weighed approximately 200 g.

The samples of each lot were divided into 10 groups of 15 samples each, as follows, and analyzed in triplicate at 0, 15, 30, 45, and 60 days (until the typical deadline of the shelf life of cold-smoked fish):

1. Control samples stored as-is (not inoculated);
2. Samples with *L. monocytogenes* mix added;
3. Samples with Sacco LAK-23 (*Lactilactobacillus sakei*) starter and a mix of *L. monocytogenes* added;
4. Samples with *Carnobacterium* spp. and mix of *L. monocytogenes* added;
5. Samples with *Lacticaseibacillus casei* (SAL 106) and a mix of *L. monocytogenes* added;
6. Samples with *Lacticaseibacillus paracasei* (SAL 211) and a mix of *L. monocytogenes* added;
7. Samples with LAK-23 (*Lactilactobacillus sakei*) added;
8. Samples with *Carnobacterium* spp. added;
9. Samples with *Lacticaseibacillus casei* (SAL 106) added; and
10. Samples with *Lacticaseibacillus paracasei* (SAL 211) added.

### Preparation of *L. monocytogenes* Suspension

The inoculum consisted of three strains of *L. monocytogenes* derived from International Culture Collections and the Collection of the Department of Agricultural Food, Environmental and Animal Sciences of the University of Udine (Di4a). In particular, the following strains were used*: L. monocytogenes* NCTC 10887 (serotype 1/2b) and *L. monocytogenes* 9Di4a (serotype 4b) from fish matrices and *L. monocytogenes* 11Di4a of human origin and responsible for invasive listeriosis. Single suspensions were prepared using a 3-day *L. monocytogenes* cultures grown at 6 ± 2°C on plate count agar (Oxoid, Italy) added to peptone water (peptone 1 g; NaCl 35 g; distilled H_2_O 1,000 ml; Aw 0.96) with a D.O. of 0.1 at 600 nm. To evaluate the concentration of each suspension, equivalent dilutions were prepared using sterile peptone water, and 0.1 ml of each dilution was surface cultured onto plates containing Palcam agar base (Oxoid, Italy). The plates were incubated at 37°C for 48 h, and the grown colonies were counted. Each suspension contained approximately 7–8 log CFU/ml.

### Preparation of the *L. monocytogenes* Suspension for the Test Samples

A cocktail (stock suspension) was prepared from suspensions containing the three different *L. monocytogenes* strains in peptone water (NaCl 3.5%; Aw 0.96; 7 log CFU/ml). The stock suspension was diluted and inoculated by spreading 1 ml onto cold-smoked sea bass fillets (final value – approximately 2 log CFU/g product).

### Starter Culture Used

One commercial and three selected starter cultures were used. All starters were bacteriocin producers, as “tested *in vitro*.”

The commercial starter culture was produced and sold by Sacco S.r.l., *Via* Alessandro Manzoni 29/a, 22,071 Cadorago, CO, Italy) and contained LAK-23 (*Lactilactobacillus sakei*, bacteriocin producer) isolated from meat product. The culture was freeze-dried, packaged in a foil pouch, and stored frozen. At the time of use, it was thawed, homogenized and diluted in sterile peptone water. To assess the culture concentration, dilutions were performed using sterile peptone water, and 0.1 ml of each dilution was inoculated into deMan Rogosa Sharpe medium (MRS, Oxoid, Italy) by the double-layer method. The plates were incubated at 37°C for 48–72 h, and the grown colonies were counted. The suspension contained, on average, approximately 11 log CFU/g.

The selected LAB starters, *Lacticaseibacillus casei* (SAL 106), *Lacticaseibacillus paracasei* (SAL 211), were isolated from milk products (Iacumin et al., 2015). *Carnobacterium maltoaromaticum* was recently isolated during a monitoring to determine the hygienic quality of “Montasio” cheese produced in Friuli. The *Lacticaseibacillus* strains were cultivated in MRS broth, and *Carnobacterium* was cultivated in TSM agar (tryptic soy medium with 5% glucose, 2% NaCl and pH 8, Oxoid, Italy). After growth, the strains were harvested by centrifugation (8,000 rpm) and then diluted in peptone water to evaluate their concentrations.

Decimal dilutions of all starter cultures were made, and then 1 ml was inoculated by spreading onto cold-smoked sea bass fillets (final value – approximately 5 log CFU/g product).

### Inoculated Samples

For each test and lot, 15 smoked sea bass samples were inoculated and analyzed in triplicate at each time point: 0, 15, 30, 45, and 60 days. Fifteen samples were stored as originally packaged and represented the controls, and the others were unpackaged and inoculated with *L. monocytogenes* alone, with all starters alone and with the starters and *L. monocytogenes* mix and then repackaged according to the technique and packaging used by the facility. All control (uninoculated) and inoculated samples were stored at 6 ± 2°C, which is the standard temperature of a supermarket refrigerator in Italy (AAVV, 2021).

### Microbiological Analysis

At the established dates, three samples from each group were subjected to microbiological analyses, which included evaluation of the total bacterial count (CBT) in Gelysate agar (gelatine sugar-free agar, Oxoid, Italy) incubated at 30°C for 48–72 h; LAB in deMan Rogosa Sharpe agar (MRS, Oxoid, Italy) incubated at 37°C for 48 h (double layer method); *Carnobacterium* in TSM agar incubated at 30°C for 2 days under anaerobiosis; yeasts and moulds in malt extract agar (MA, Oxoid, Italy) incubated at 25°C for 72–96 h; total coliforms and faecal coliforms in violet red bile lactose agar (VRBLA, Oxoid, Italy) incubated, respectively, at 37 and 44°C for 24 h; coagulase-positive staphylococci in Baird–Parker agar medium (BP, Oxoid, Italy) with egg yolk tellurite emulsion added (Oxoid, Italy) incubated at 35°C for 24–48 h and confirmed by the coagulase test; sulfite-reducing Clostridia in differential reinforced clostridial medium (DRCM, VWR, United States) incubated at 37°C for 24–48 h in a jar prepared for anaerobic reaction with a gas-packing anaerobic system (BBL, Becton Dickinson, United States); *L. monocytogenes* detected and quantified according to the ISO method (ISO 11290-1:1996 Adm.1:2004, n.d.) and *Salmonella* spp. detected and quantified according to the ISO method (ISO 6579-1: 2002 Cor.1:2004, n.d.). To confirm the growth of the starters, five colonies were collected from deMan Rogosa Sharpe plates and TSM agar and then identified by the methods reported in Iacumin et al. (2009).

### Physicochemical Analysis

The control samples (1) and samples inoculated with the starters (7, 8, 9, and 10) alone were also subjected to chemical–physical analyses. In particular, the pH was at three different points using a pH metre (Basic 20, Crison Instruments, Spain) by inserting the probe directly into the product. The water activity (Aw) was measured with an Aqua Lab 4 TE (Decagon Devices, United States). Humidity was measured according to the AOAC (1990), and NaCl and TVB-N (total volatile basic nitrogen) were measured according to Pearson (1973). WPS (water-phase salt) was determined according to the formula according to Huss et al. (1997):


$$\mathrm{WPS}\;\left(g/100\;\;\mathrm{ml}\right)=\frac{\mathrm{salt}\;\;\mathrm{content}\left(\mathrm{in}\;\;g\;\;\mathrm{per}\;\;100\;\;g\right)}{\begin{array}{l} \mathrm{moisture content}\;\left(\mathrm{in}\;\mathrm{ml}\;\mathrm{per}\;100\;\;g\right)\times \\ \mathrm{salt content}\;\left(\mathrm{in}\;g\;\mathrm{per}\;100\;g\right) \end{array}}\times 100$$

Thiobarbituric acid-reactive substances (TBARS) were determined according to Ke et al. (1984). The pH values were detected at determined at 0, 15, 30, 45, and 60 days, while the other physicochemical parameter at the beginning (0 day) and at the end (60 days) of the shelf life. At each time point, the analyses were performed on three samples.

### Statistical Analysis

Data were analyzed using Statistica 7.0 version. 8 software (Statsoft Inc., 2008). The values of the different parameters were compared by one-way analysis of variance and the means were then compared using Tukey’s honest significance test. Differences were considered significant at *p* < 0.05.

### Sensory Analysis

Sensory analysis was performed by 20 nonprofessional trained tasters. Ten additional samples of treatments 1 and 7 were evaluated by tasters who were asked to evaluate the influence of the LAB starter on the organoleptic and sensory characteristics of the products. Sensory analysis was performed based on the triangle test (ISO 4120:2004, n.d.; triangle test). In short, 20 nonprofessional trained tasters were presented with three products, two of which were identical. The choice of nonprofessional tasters was mandatory because they represent typical consumers. The tasters were asked whether they understood the examined differences, and in the case of differences they were asked to specify the type, for example, color, texture, bouquet, flavor, or smell. Among the samples with starter added, only seven were tested, considering that the LAK-23 was the only strain inhibiting the growth of *L. monocytogenes*.

## Results

### Monitoring *Listeria monocytogenes* in Cold-Smoked Fish

The survey highlighted the presence of *L. monocytogenes* in 3.4% of all 440 samples analyzed (Table 1). However, among the individual products, *L. monocytogenes* was isolated in 6% of smoked salmon samples and 4% of smoked sea bass fillets (new product). In contrast, it was not isolated from smoked trout. *Listeria monocytogenes* was almost always determined at the level of presence in 25 g. In only one case, which was a pack of smoked salmon, its presence was at the level of 40 CFU/g (Table 2).

**Table 1:** *Listeria monocytogenes* in marketed cold-smoked fish.

Product	N. samples	N. positive samples/%	*L. monocytogenes* isolates
Salmon	150	9/6	40
Trout	140	0/0	0
Sea bass^*^	150	6/4	25
Total	440	15/3.4	65

* New product – not marketed.

**Table 2:** Concentration of *L. monocytogenes* in cold-smoked fish.

Range	Salmon	Trout	Sea bass
Presence/25 g	8	0	6
1–100 UFC/g	1^*^	0	0
100–1000 UFC/g	0	0	0
N. positive	9	0	6

* 40 CFU/g.

Sixty-five isolated *L. monocytogenes* were serotyped using commercial sera. As shown in Table 3, 42 strains belonged to serotype 1/2c, 14 to serotype 4b, 6 to serotype 1/2b, and 3 to serotype 1/2a. The serotypes observed represent 95% of strains isolated from food and clinical isolates.

**Table 3:** Serotypes of *L. monocytogenes* in cold-smoked fish.

Serotypes	Salmon	Trout	Sea bass
1/2a	0	0	3
1/2b	3	0	3
1/2c	28	0	14
4b	9	0	5
Total	40	0	25

Finally, in the microbiological analyses, total and fecal coliforms, coagulase-positive Staphylococci and sulfite-reducing clostridia were not quantified (<5 CFU/g), and *Salmonella* spp. was always absent in 25 g.

### Physicochemical Analyses

Physicochemical analyses was performed on all samples. The values of samples with starters added were grouped into two group. The first group includes the samples added with *Carnobacterium*, showing a light pH decreasing no longer significant according to the SD values between 0 and 60 days (Table 4). The second group includes the samples added with *Lacticaseibacillus casei* 211, *Lacticaseibacillus paracasei* 106, and *Lactilactibacillus sakei* (LAK-23). In this case, it was observed a slight decreases between 0 and 30 days, and a significant drop between 30 and 60 days (*p* < 0.05). Therefore, despite the development of the added starter, the decrease in pH was only 0.1 units.

**Table 4:** pH evolution in cold-smoked sea bass stored at 6 ± 2°C with and without LAB starter cultures added.

Days	Control trial	*Carnobacterium*	*Lb. casei* 211	*Lb. paracasei* 106	*LAK-23*
0	5.94 ± 0.05^a^	5.94 ± 0.05^a^	5.94 ± 0.05^a^	5.94 ± 0.05^a^	5.9 ± 0.05^a^
15	5.95 ± 0.03^a^	5.97 ± 0.02^a^	5.93 ± 0.02^a^	5.99 ± 0.02^a^	5.80 ± 0.02^b^
30	5.96 ± 0.05^a^	5.96 ± 0.04^a^	5.91 ± 0.07^a^	5.96 ± 0.02^a^	5.90 ± 0.01^a^
45	5.99 ± 0.05^a^	5.91 ± 0.06^a^	5.81 ± 0.03^b^	5.83 ± 0.03^b^	5.80 ± 0.07^b^
60	5.95 ± 0.03^a^	5.90 ± 0.03^a^	5.81 ± 0.07^b^	5.84 ± 0.13^a,b^	5.80 ± 0.08^b^

All data are represented as mean ± SD; means with same letters, following the columns, are not significantly different (*p* > 0.05). LAK-23, Lactilactobacillus sakei.

The values of moisture, NaCl and Aw did not change significantly (*p* > 0.05) in either the controls or samples supplemented with starter (Table 5). The Aw remained within the range of 0.970 and 0.971. In all samples, no significant difference was observed over time (*p* > 0.05). However, it is thought that the small differences in Aw observed at the various sampling points were probably due to variation among the samples and were not correlated to real water loss. The moisture content remained fairly constant over time. It exhibited values between 59.21 and 59.55%, and the observed differences were due to the different samples analyzed rather than the absorption or loss of moisture (Table 5).

**Table 5:** Physicochemical parameters in cold-smoked sea bass stored at 6 ± 2°C with^*^ or without starters added.

Parameters	Days			
	0	0^*^	60	60^*^
% Moisture	59.21 ± 0.15^a^	59.51 ± 0.44^a^	59.60 ± 0.31^b^	59.55 ± 0.25^b^
% NaCl	3.3 ± 0.11^a^	3.2 ± 0.60^a^	3.0 ± 0.21^a^	3.1 ± 0.11^a^
Aw	0.970 ± 0.002^a^	0.970 ± 0.001^a^	0.971 ± 0.009^a^	0.971 ± 0.002^a^
% WPS	5.2 ± 0.03^b^	5.0 ± 0.11^a^	5.1 ± 0.5^c^	5.1 ± 0.18^d^
TVB-N mg N/100 g	30.2 ± 0.11^a^	33.05 ± 1.00^b^	35.50 ± 0.28^c^	35.00 ± 0.28^c^
TBARS nmol/g	5.5 ± 0.2^a^	6.1 ± 0.2^b^	6.6 ± 0.3^c^	6.4 ± 0.5^c^

WPS, water salt phase; TVB-N, total volatile nitrogen; TBARS, thiobarbituric acid – reactive substances-malonaldehyde index. Data represent the means ± SDs of the total samples; means with the same letters, follow the lines and considering each parameters, are not significantly different (*p* < 0.05).

The WPS value varied according to the tested samples, but it was over 5.0% in all samples (Table 5). These values are acceptable for cold-smoked fish. In this study, the salt and WPS values varied over the storage period without showing a specific trend, indicating that the observed differences were due only to variability among the samples. The salt content was influenced by the variability of the samples and the salting procedure. For these reasons, the observed decreases cannot be considered trends but were due to random heterogeneity in the samples.

Additionally, the average values of TVB-N and TBARS (Table 5) were largely acceptable. The smoked sea bass showed average values of TVB-N of 30.2 and 33.05 mg N/100 g immediately after packaging, which and evolved to 35.50 and 35.00 mg N/100 g at the end of the shelf life. Indeed, significant increases were observed over time, which was limited to 3–5 mg N/100 g. The TBARS increased during storage but remained at the maximum level of 6.6 nmol malonaldehyde/g at the end of storage (60 days). However, over time, an increase in this parameter was observed. At 0 day, the TBARS values were 5.5 and 6.1 nmol malonaldehyde/g, and then they increased significantly, reaching levels of 6.4–6.6 nmol malonaldehyde/g (*p* > 0.05).

### Microbiological Analysis

*Lacticaseibacillus casei 211*, *Lacticaseibacillus paracasei 106*, *Carnobacterium maltoaromaticum*, and *Lactilactobacillus sakei* (LAK-23) were the bioprotective cultures used in this work against *L. monocytogenes*. Their use was based on the qualified presumption of safety (QPS), which indicates the safety status of microorganisms intentionally used in food and the feed chain, certifying that they do not pose a risk to human or animal health based on the scientific literature (EFSA BIOHAZ Panel et al., 2020).

**Figure 1:**
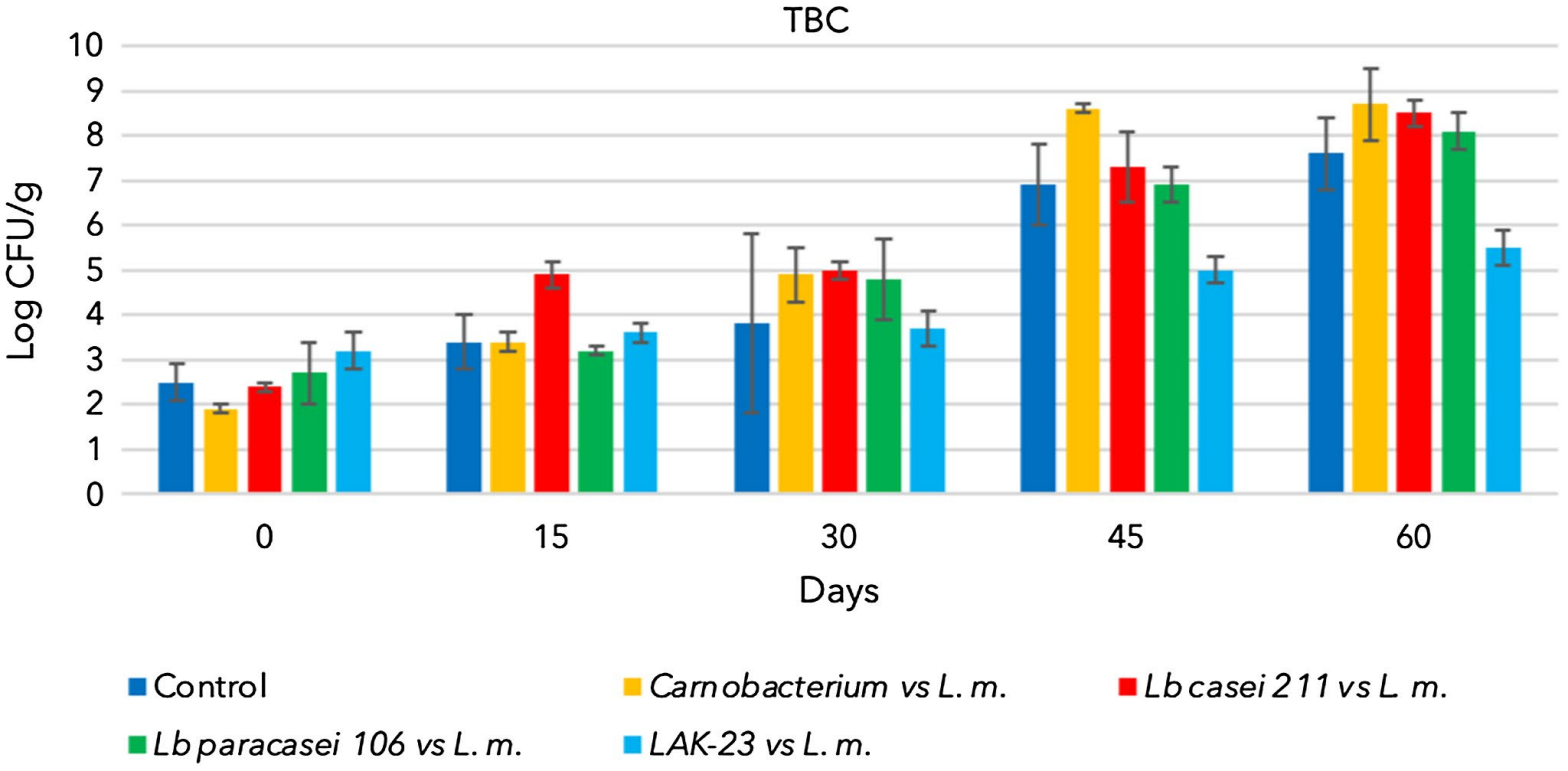
Evolution of total bacterial count in cold-smoked sea bass with or without bioprotective starter added, stored at 6 ± 2°C.

**Figure 2:**
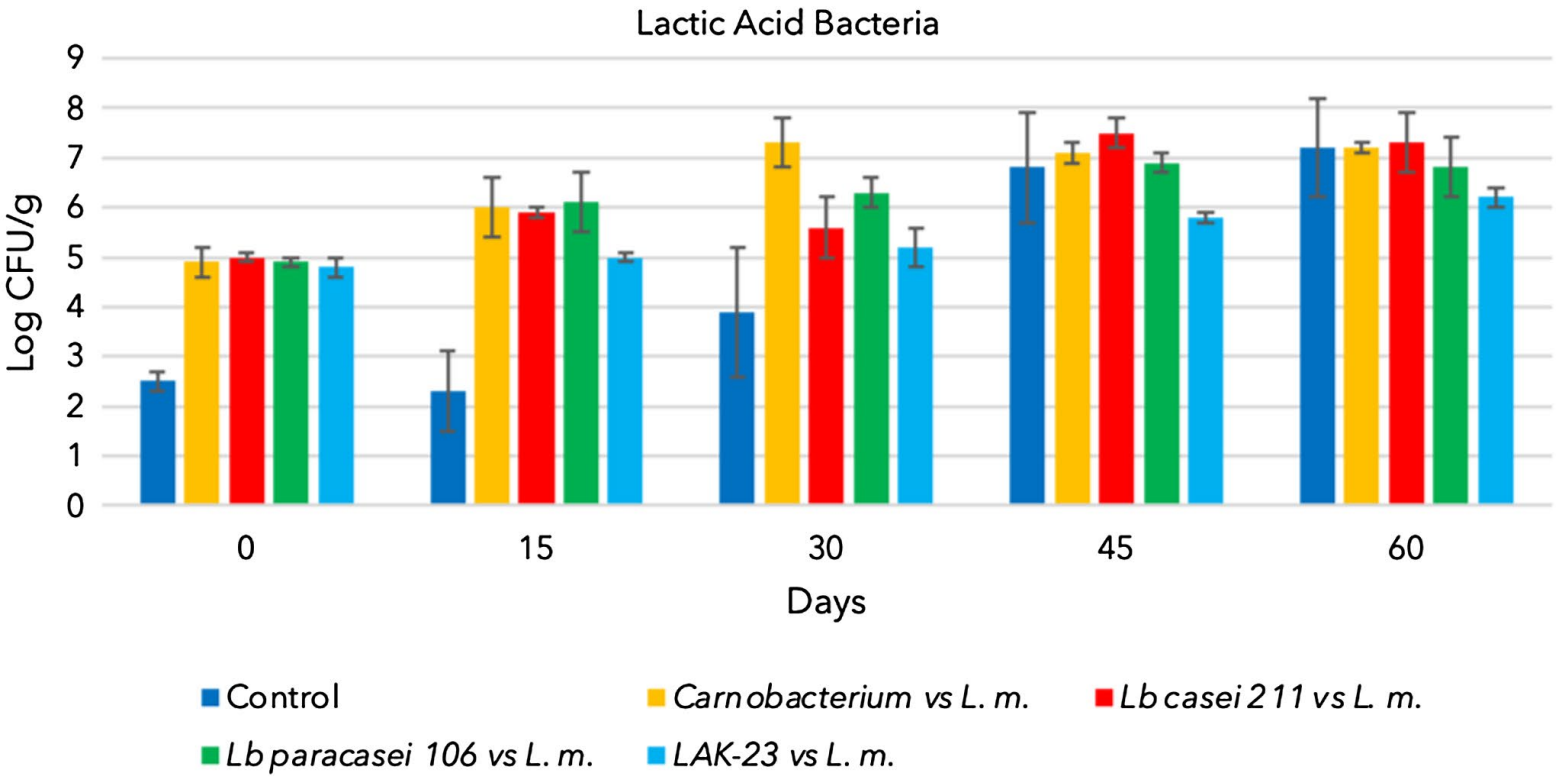
Evolution of lactic acid bacteria in cold-smoked sea bass with or without bioprotective starter added, stored at 6 ± 2°C.

All starter cultures and *L. monocytogenes* grew during storage when they were separately inoculated and reached 7–8 log CFU/g (data not shown). Additionally, natural TBC and LAB (control samples) grew and reached approximately 7 log CFU/g at the end of sampling (Figures 1, 2). Consequently, cold-smoked fish represent a good substrate for microorganism growth. Indeed, in the control samples, TBC grew constantly during the entire period, as well as indigenous LAB (Figures 1, 2). The latter are lactic acid bacteria, which usually contaminate substrates and increase in number during the shelf lives of products. These LAB do not present a hazard to consumers, but sometimes, heterofermentative LAB can influence the quality of final products. Other analyses were also performed to confirm that anaerobes and coliforms were not present in the samples. Finally, as expected, in the control samples, the presence of *L. monocytogenes* was below the detection limit (Absence in 25 g).

**Figure 3:**
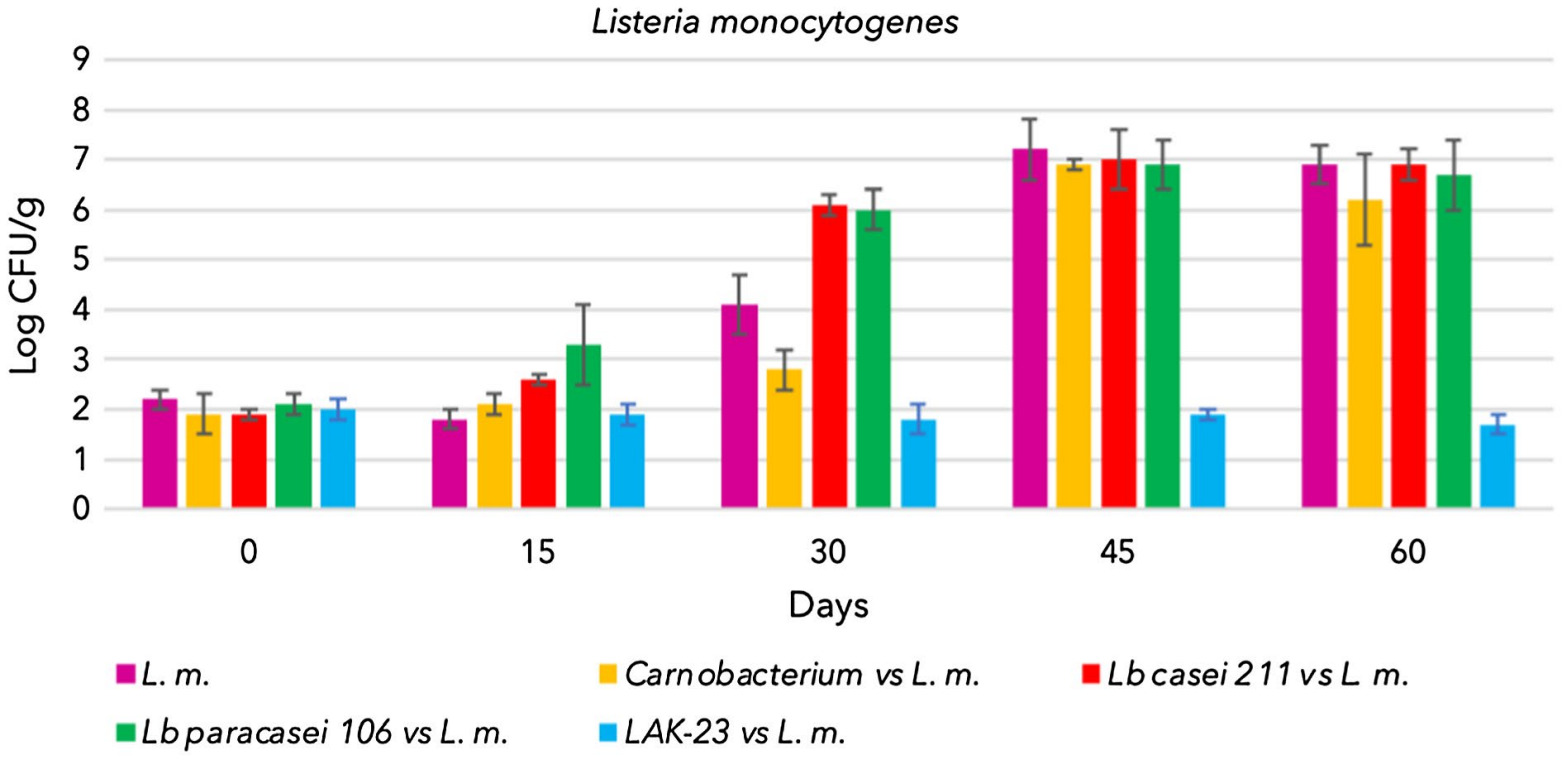
Evolution of *Listeria monocytogenes* intentionally inoculated in cold-smoked sea bass with or without bioprotective starter added, stored at 6 ± 2°C.

In addition, the presence of autochthonous LAB did not influence the growth of *L. monocytogenes*. In samples in which only the pathogen was inoculated, indigenous LAB increased but did not influence the growth of *L. monocytogenes*, which reached high concentrations, as illustrated in Figure 3. Cold-smoked sea bass is indeed a suitable substrate upon which *Listeria* can grow despite the temperature of storage, presence of smoking compounds, and high salt concentration (3.5%).

The trends from trials of *Lacticaseibacillus casei* 211, *Lacticaseibacillus paracasei* 106 and *Carnobacterium* spp. coinoculated with *L. monocytogenes* are shown in Figures 1–3.

As shown TBC and the bioprotective starter grew during the whole storage time. Unfortunately, he coinoculated *L. monocytogenes* also grew, reaching hazardous level (8 log CFU/g). Consequently data demonstrated that of *Lacticaseibacillus casei* 211, *Lacticaseibacillus paracasei* 106 and *Carnobacterium maltoaromaticum* were not able to limit the growth of the pathogen, which could have become a serious risk to health (Figure 3).

Figure 2 shows the evolution of *Lacticaseibacillus paracasei* 106 coinoculated with *L. monocytogenes*. Even in these trials, TBC evolved over time, increasing from approximately 2.5 log CFU/g to 8 log CFU/g (Figure 1). *Lacticaseibacillus paracasei* 106 increased significantly from 0 to 60 days, reaching approximately 7 log CFU/g (Figure 2). Coinoculated *L. monocytogenes* showed significant increases until 60 days, prevailing over the starter culture through 45 days. Then, at 60 days, its concentration was less than that of *Lacticaseibacillus paracasei* 106 (Figure 3).

Figures 2, 3 highlight the evolution of the microbial population of cold-smoked sea bass fillets intentionally inoculated with the starter LAK-23 and *L. monocytogenes*. The starter developed (Figure 2) and markedly inhibited inoculated *Listeria*. In fact, the concentration of the *L. monocytogenes* mix remained almost constant over time (Figure 3), although slight decreases were noted at 45 and 60 days, which did not appear significant considering the large SD (*p* > 0.05). Therefore, the starter used was effective, and although it did not reduce or completely eliminate the inoculated strains of *L. monocytogenes*, it still prevented their growth. Additionally, also TBC grew in samples inoculated with LAK-23 (Figure 1). In fact, it reached values of 6 log CFU/g at the end of the shelf life.

Finally, the growth of the added starters in samples inoculated with *L. monocytogenes* was confirmed by the identification of the colonies isolated from MRS and TSM agar (data not shown).

### Sensory Analysis of Cold-Smoked Sea Bass Fillets

The samples were brought to environmental temperature immediately before the administration and checked for the presence of atypical odors and flavors, white or viscous patinas, slime, discoloration or browning. Neither the samples nor the controls were positive for the previously described parameters and consequently underwent the sensory evaluation.

The sensory acceptability of samples with or without starter was determined by the triangular test. No sensory differences were perceived between samples inoculated with LAK-23 and control samples (uninoculated). In fact, neither group of samples was recognized as different. From the comparison of the samples under analysis, it emerged that they belonged to a single sample. Therefore, the starter did not profoundly modify the sensory characteristics of the product to which it was added.

## Discussion

*Listeria monocytogenes* was determined in different smoked fish (salmon, trout, and sea bass) at the level of presence in 25 g. In only one case, which was a pack of smoked salmon, was its presence at the level of 40 CFU/g. These percentages and concentrations are clearly lower than those determined by other authors (Medrala et al., 2003; Garrido et al., 2009; Dass et al., 2010, 2011; Kramarenko et al., 2013; Tocmo et al., 2014). In particular, Miettinen et al. (1999) showed the presence of *L. monocytogenes* at the level of 1.9 × 10^5^ CFU/g in smoked fish that caused gastroenteritis. However, in another work, it appeared that in smoked salmon, a concentration of 45 CFU/g was sufficient to trigger listeriosis in at-risk individuals (Marozzi et al., 2015). The observed small changes in the samples examined could be due to the short time elapsed between production and analysis. All samples were analyzed within 15–20 days from production and 40–45 days from expiry. Johansson et al. (1999) reported completely different results, having observed that *L. monocytogenes* was present in up to 20% of RTE smoked fish products in Finland and that its concentration was almost always >100 CFU/g.

The serotypes observed include 95% of strains isolated from foods and patients suffering from listeriosis. In fact, over time, a clear prevalence of serotypes 1/2a, 1/2b, 1/2c, and 4b has been observed in various foods (Comi et al., 1992; Doumith et al., 2004). However, unlike the results observed by the aforementioned authors, a dominance of serotype 1/2c was noted in monitoring during this experiment, and this serotype is considered not very virulent (Pinner et al., 1992). In fact, the literature shows that the most common strains involved in 90% of listeriosis from smoked fish products belong to serotypes ½a, ½b, and 4b (Comi et al., 1992; Doumith et al., 2004). Moreover, subdivision of the serotypes of *L. monocytogenes* on the basis of levels of virulence has identified four evolutionary lines (I, II, III, and IV) with different but overlapping ecological niches (Doumith et al., 2004; Orsi et al., 2011). Most *L. monocytogenes* isolates appear to belong to lines I and II, which host the serotypes most commonly associated with human clinical cases, including serotype ½a (line II) and serotypes ½b and 4b (line I). Strains of line II are common in foods, appear to be widespread in natural and farm environments, and are also commonly isolated from cases of animal listeriosis and sporadic human clinical cases (Comi et al., 1992; Miettinen et al., 1999; Doumith et al., 2004; Orsi et al., 2011). However, it appears that most outbreaks of human listeriosis are associated with isolates belonging to line I (Miettinen et al., 1999; Orsi et al., 2011). Furthermore, numerous studies indicate that, in many countries, strains of line I are more present among human isolates than strains of line II. Strains of lines III and IV, on the other hand, are rare and mainly isolated from animal sources. On the basis of reporting and serotypes belonging to the different lines, it can be concluded that the majority of the isolated strains belong to serotype 1/2c, which seems to be considered less virulent compared to the more widespread and virulent serotypes 1/2a, 1/2b, and 4b (Comi et al., 1992; Pinner et al., 1992; Doumith et al., 2004; Orsi et al., 2011).

Spoilage and pathogenic microorganisms were not detected, considering that total and fecal coliforms, coagulase-positive Staphylococci and sulfite-reducing clostridia were not quantified (<5 CFU/g) and *Salmonella* spp. was always absent in 25 g.

The physicochemical parameters of the cold-smoked sea bass indicated that the moisture and salt concentrations did not change over time, and the minimal variations observed were due to variation among the samples, which were different at each sampling time. In addition, as expected, the pH varied at a level of 0.1 units in samples supplemented with starters, including *Lb. paracasei* 106, *Lb. casei* 211 and LAK-23 (*Lb. sakei*). LAB produce lactic acid and other acids, which decrease the pH of food (Comi, 2016). The above species used as starter cultures are recognized as able to produce acids, and consequently, their growth produced a sensible decrease in pH.

The values of salt and WPS were adequate for cold-smoked fish. Indeed, regarding the salt content and WPS, the literature underlines the importance of considering a WPS value of 3.5% as the minimum value capable of preventing the growth of *Clostridium botulinum* type E, which is psychotrophic, at the lower storage temperature of 4.4°C (Centre for Food Safety and Applied Nutrition, 2001). In this study, the salt and WPS values varied over the storage period without showing specific trends, indicating that the observed differences depended only on variability among the samples and the salting procedure. For these reasons, the observed decreases cannot be considered trends but were due to random heterogeneity in the samples. The observed WPS values, which were closely related to the percentage of salt present, ranged from 4.1 to 5.2%, and these values largely satisfy the limits set by the Centre for Food Safety and Applied Nutrition (2001). These values can ensure health safety throughout the storage time and up to the time of consumption, as demonstrated by different authors, who found WPS values over 4% in Italian and French smoked salmon (Hespe et al., 2004; Cornu et al., 2006; Bernardi et al., 2009, 2011). It is likely that the high WPS value may have influenced the growth of CBT and LAB in the tested smoked sea bass samples. In fact, these values are clearly lower than those observed by Bernardi et al. (2009) in smoked salmon.

The average values of TVB-N in the samples were largely acceptable, as they were within the standard parameters of smoked seafood, although Chilean authorities (Sernapesca, 1996) have set a limit of 30 mg N/100 g for cold-smoked salmon. Considering the literature data, such a low value seems unattainable (Buchrieser et al., 1993). The smoked sea bass showed average values of TVB-N at the end of storage of 35.50 (samples with starter added) and 35.00 mg N/100 g (control). Indeed, a significant increase was observed over time. However, the increase was limited to 3–5 mg N/100 g. The final values of TVB-N were close to 35 mg N/100 g and clearly lower than the maximum values (40 mg N/100 g) proposed by Cantoni et al. (1993) for smoked salmon. In any case, the data regarding TVB-N are different from those obtained by Bernardi et al. (2009), who observed TVB-N values of 49.8 mg N/100 in Italian smoked salmon at the end of shelf life. It must be noted that the same authors showed initial TVB-N values of approximately 38.2 mg N/100 g, which are values significantly higher than those observed at time 0 in smoked sea bass fillets. Additionally, Leroi et al. (1996, 1998) observed TVB-N values equal to 52.8 mg N/100 g, and these values are significantly higher than those observed here.

According to several authors (Ke et al., 1984; Man and Ramadas, 1998), food products are not rancid when TBARS values are <8 nmol malonaldehyde/g product, slightly rancid when TBARS are between 9 and 20 nmol malonaldehyde/g, and rancid and unacceptable when TBARS are >21 nmol malonaldehyde/g. Consequently, all samples of cold-smoked sea bass were considered acceptable, given the low TBARS values. Moreover, the tasters in the panel did not perceive hints of rancidity.

Cold-smoked seabass is a fishery product that, at the end of production, is usually free from *L. monocytogenes*. However, in the case of contamination in the production chain, this pathogen can multiply during the product shelf life. In fact, cold-smoked seabass shows the ideal growth characteristics for *L. monocytogenes*: a pH > 5, as shown by the physicochemical analysis carried out in this study, and an Aw of approximately 0.96, as also reported in a study conducted by Aymerich et al. (2019) in cold-smoked salmon. Even the storage temperature plays a fundamental role. In general, cold-smoked sea bass is a ready-to-eat (RTE) product that needs to be stored constantly at +4°C. However, it is possible that during its shelf life, the product could face thermal abuse, further supporting the growth of *L. monocytogenes*. In our study, this condition was tested by keeping the seabass at 6 ± 2°C for the entire period (60 days).

In addition to the pH, Aw and temperature, the salt content is also important. Cold-smoked seabass, similar to other similar products, has a salt content of 3.5% (NaCl), but this level is not enough to limit *L. monocytogenes* growth since the pathogen can easily multiply in matrices containing up to 10–12% NaCl. As demonstrated by Vaz-Velho et al. (2005), variation in the salt content of smoked fish samples is unlikely to affect *L. monocytogenes* growth.

Therefore, the use of the selected LAB as bioprotective starter cultures presents a valid way to prevent or limit the development of pathogens such as *L. monocytogenes*. *L. monocytogenes* was reduced in whole milk by different *Lactobacillus* strains (Garcia et al., 2019) and by *Carnobacterium piscicola* in refrigerated food (Campos et al., 1997).

Nevertheless, in this research, the starter cultures acted differently. The two LAB cultures (*Lb casei* 211 and *Lb. paracasei* 106) did not have any effect against *L. monocytogenes* since the pathogen reached high concentrations at 30 days. Indeed, *Carnobacterium maltoaromaticum* showed minimal growth control against *L. monocytogenes*, which increased by only 1 log CFU/g within 30 days. However, the effect was not sufficient when considering the whole period (60 days), in which the pathogen concentration reached hazardous levels (7 log CFU/g). The failure to compete with *L. monocytogenes* in the long term through the production of bacteriocins could be attributed to many different factors. One could be the presence of food components in the substrate that might affect bacteriocin production and activity (Aasen et al., 2003). In this regard, Dos Reis et al. (2011) considered the influence of the food matrix on *Carnobacterium* important, as the use of natural preservatives in some cases inhibited bacteriocin production, and Vaz-Velho et al. (2005) noticed that bacteriocin activity was reduced, particularly after the smoking process in cold-smoked salmon and trout. In addition, Aymerich et al. (2019) showed that a *Carnobacterium* strain exhibited antilisterial activity *in vitro* assays but did not exert a significant antilisterial effect in all tested products. For this reason, the food matrix and smoking phase could have influenced the ability of the three starters (*Lb. casei* 211, *Lb. paracasei* 106 and *Carnobacterium maltoaromaticum*) to produce bacteriocins, which could explain why *L. monocytogenes* rose to very high concentrations.

On the other hand, LAK-23, which was *Lactilactobacillus sakei*, was able to inhibit *L. monocytogenes* growth in cold-smoked sea bass. In particular, at the end of storage, *L. monocytogenes* was found at the level of the initial inoculum (2 log CFU/g). This strain is used either as a starter to promote food ripening or as a bioprotective culture. In particular, considering its fast growth at storage temperatures and bacteriocin production, it was suggested for use in processed meat, and in particular, fish products (Sacco, https://www.saccosystem.com, accession 01/10/2021).

Various studies have demonstrated the role of LAB (*Lactobacillus*, *Carnobacterium*, and *Enterococcus*) and bacteriocins in inhibiting *L. monocytogenes* in smoked fish products (Nilsson et al., 1997; Leroi et al., 1998, 2015; Richard et al., 2004; Weiss and Hammes, 2006; Tomé et al., 2008; Concha-Meyer et al., 2011; Rotariu et al., 2014). However, the use of bacteriocins such as nisin and sakacin P was effective against *L. monocytogenes* only in the short term, and consequently, the direct use of bacteriocin-producing starters or the addition of other antimicrobials such as organic acids or essential oils has been suggested (Vaz-Velho et al., 2005; Tomé et al., 2008; Tocmo et al., 2014). Indeed, the application of nisin showed a listeriostatic effect in cold-smoked rainbow trout for only 3 days (Nykänen et al., 2000). However, upon combining nisin and lactate, the *L. monocytogenes* count showed a 2-log reduction after 17 days of storage (Nykänen et al., 2000). In another study, the use of sakacin P and *L. sakei* Lb790 resulted in a 2-log reduction in *L. monocytogenes* count after 28 days (Katla et al., 2001). These studies indicate that the potential synergistic effects of combining bacteriocins with other hurdles can extend the duration of inhibition. However, better results have been obtained using bioprotective starters directly, as demonstrated by Aymerich et al. (2019). These authors evaluated three potential bacterial strains against *L. monocytogenes* in smoked salmon with different physicochemical characteristics (fat concentration, moisture and acetic acid). Among the strains used, two were bacteriocin producers isolated directly from smoked salmon and identified as *Lactobacillus curvatus* and *Carnobacterium maltaromaticum*, while the third was of meat origin and identified as *Lactobacillus sakei* CTC494, which is a bacteriocin producer. The data demonstrated that *L. sakei* CTC494 inhibited the growth of *L. monocytogenes* after 21 days of storage at 8°C in all products tested, while *L. curvatus* CTC1742 only limited the growth of the pathogen (<2 log increase). The efficacy of *C. maltaromaticum* CTC1741 depended on the type of product. In fact, it limited the growth of the pathogen only in one type of smoked salmon. The results obtained by Aymerich et al. (2019) suggest that *L. sakei* CTC494, despite having been isolated from meat, can potentially be used as a bioprotective agent to improve the food safety of cold-smoked salmon. Likewise, *Lactilactobacillus sakei* (LAK23), despite it was isolated from meat products, has been demonstrated able to inhibit *L. monocytogenes* growth in cold-smoked sea bass.

The presence of intentionally inoculated selected LAB can also have other positive effects. As illustrated in the microbiological analyses, indigenous LAB could also grow in the product, achieving high concentrations (approximately 7–8 log CFU/g). Inoculation with selected starter cultures can prevent the development of indigenous microbial populations and, consequently, limit the release of secondary products such as CO_2_, organic acids, and ethanol. These substances are not considered to positive affect the quality of RTE sea bass, as they can cause alteration of sensory characteristics (odor, color, and texture) and packaging swelling. As cold-smoked sea bass is packed under vacuum, gas production entails the loss of vacuum. Many studies have observed that some organoleptic characteristics are less altered in products treated with LAB compared with untreated samples and that the use of selected starter cultures leads to an improvement in sensory characteristics and antioxidant capacities (Udomsil et al., 2011; Anacarso et al., 2013; Li et al., 2020; Zhou et al., 2021). This is due to LAB competition with endogenous microbiota, such as spoilage bacteria. LAB are also able to produce positive compounds, such as volatiles, and to reduce negative compounds (Huang et al., 2021). These capabilities provide quality preservation and improvement and shelf life extension. In addition, the utilization of multibacterial mixed fermentation can improve food characteristics and produce different positive compounds than single-bacterial fermentation (Huang et al., 2021).

## Conclusion

The results obtained confirm that *L. monocytogenes* can be isolated from cold-smoked fish products. These products are characterized by a physicochemical composition capable of supporting its growth. They have pH values greater than 5.8 units, Aw values greater than or equal to 0.97 and humidity values equal to 59–60%. Usually, initial contamination, which is derived from the raw materials, humans and the processing environment, is limited to a few cells per g of product. Processing technology based on salting, cold smoking, vacuum packaging and refrigeration is not able to eliminate the hazard represented by *L. monocytogenes*. The results obtained show that the Aw values of cold-smoked sea bass were never lower than 0.97 and that the pH did not drop below 5.0 units. Consequently, the product can support the growth of *L. monocytogenes*. To remedy this, the use of bioprotective starters was suggested. The LAB contained in the starter grew throughout the storage period and did not block the growth of the pathogen. Only LAK-23 was able to stop the increase in the *L. monocytogenes* load, which remained at the inoculum level until the end of storage. Thus, its use as bioprotective agent is suggested. Therefore, although cold-smoked sea bass does not have a pH ≤ 4.4 and Aw ≤ 0.92 or pH ≤ 5.0 and Aw ≤ 0.94, as stated in Regolamento (CE) n. 2073/2005 (n.d.), the results scientifically affirm that these products with added bioprotective starters are not a favorable medium for the growth of *L. monocytogenes*. Consequently, they could easily fall into category 1.3 (ready-to-eat foods that do not constitute a favorable medium for the growth of *L. monocytogenes*, other than those intended for infants and special medical purposes), in which a maximum concentration of *L. monocytogenes* of 100 CFU/g is allowed. In addition the sensory analysis demonstrated that the use of starters does not depreciate nor mischaracterize cold-smoked sea bass.

## Data Availability Statement

The raw data supporting the conclusions of this article will be made available by the authors, without undue reservation.

## Author Contributions

LI, GCa, MP, MB, and GCo contributed equally to the planning, the testing, reporting findings and discussion of the work. All authors have read and agreed to the published version of the manuscript.

## Funding

This work was supported by Interreg Italia-Croazia, AdriaAquanet Priority Axis:Blue Innovation. AdriAquaNet ID 10045161.

## Conflict of Interest

None of the authors of this paper has a financial or personal relationship with other people or organizations that could inappropriately influence or bias the content of the paper.

## Publisher’s Note

All claims expressed in this article are solely those of the authors and do not necessarily represent those of their affiliated organizations, or those of the publisher, the editors and the reviewers. Any product that may be evaluated in this article, or claim that may be made by its manufacturer, is not guaranteed or endorsed by the publisher.
